# Salivary Calculus

**Published:** 1858-04

**Authors:** A. C. Castle


### ARTICLE VI.

### *Salivary Calculus.* By A. C. CASTLE, M. D.

SALIVARY calculus—the offensive deposit upon and around the teeth—has of late years received a large share of practical attention from dentists, authors, and others: but not to that extent which its peculiar importance demands. Its character has, indeed, been the subject of many theories, some of which, if not ridiculous, are humorous, from their very absurdity; whilst others have been deserving respect. Some authors! have supposed this substance to be "*exhaled*" from the gums; others, quite as unique in their theory, think that it is built up by animalculæ—(and, who, even go so far as to illustrate their character)—similar to the the construction, or rather, building up of "coral reefs;" and numerous dental practitioners, in common with popular opinion, ascribe its presence to the absence of cleanliness.

It is computed that, from twenty-four to twenty-eight ounces of saliva, in quantity, is secreted into the mouth every *twenty-four hours.* It will, therefore, be observed, that the elements of the saliva, in the process of digestion, exert a more important position and influence than its *apparent* insignificance would lead the ordinary observer to suppose. Its elementary or constituent principles, in health, are mucilage, albumen, iron phosphate, and oxalate of lime, and phosphate of ammonia and magnesia, and alkaline in its nature. In disease, especially in aphthous children, and those under the influence of scrofulous, phthisical, and gout disease, the saliva is more or less acidified.

Chemical analysis shows the following composition of the soluble and insoluble matter which forms the basis of *salivary calculus.*

The constituents soluble in water—

| | |
|---|---|
| Tribasic phosphate of soda, (3 Na O, PO s,) | 28.122 |
| Chloride of sodium and potassium, (s) . | 61.930 |
| Sulphate of soda, . . . . . | 2.315 |

The constituents insoluble in water—

| | |
|---|---|
| Phosphate of lime. | |
| " of magnesia. | |
| " of peroxyd of iron, . . . | 5.509 |
| Loss, . . . . . . . . | 2.124 |

The chemical composition of the substance of the teeth is as follows:

*The ivory or bone of the teeth.*

| | |
|---|---|
| Phosphate of lime, | 61.95 |
| Fluate of lime, | 1.10 |
| Silica, | 1.00 |
| Carbonate of lime, | 5.30 |
| Phosphate of magnesia, | 1.25 |
| Soda and chloride of sodium, | 1.40 |
| Cartilage (animal matter) and water, | 28.00 |

*The enamel of the teeth.*

| | |
|---|---|
| Phosphate of lime, | 85.3 |
| Fluate of lime, | 3.00 |
| Silica, | 1.75 |
| Carbonate of lime, | 8.00 |
| Phosphate of magnesia, | 1.00 |
| Animal matter and water, | 1.22 |

*Ashes of the human blood.* (BERZELIUS.)

| | |
|---|---|
| Tribasic phosphate of soda, | 22.1 |
| Chloride sodium, | 54.769 |
| " potassium, | 4.116 |
| Sulphate of soda, | 2.461 |
| Phosphate of lime, | 3.636 |
| " of magnesia, | 0.769 |
| Oxyd of iron, with phosphate of iron, | 10.77 |

*The fœces of adults.* (BERZELIUS.)

| | |
|---|---|
| Tribasic phosphate of soda, | 2.633 |
| Chloride of sodium and alkaline sulphates, | 1.367 |
| Silica, | 7.940 |
| Sulphate of lime, | 4.530 |
| Phosphate of lime, " of magnesia, } | 80.373 |
| Phosphate of peroxyd of iron, | 2.090 |

Here, in the latter, are found 4.000 soluble, and 94.933 insoluble salts.

*Pulmonary Mucus.* (NAPE.)

| | |
|---|---|
| Water, | 955.320 |
| Solid residue, | 44.480 |
| Mucin, } | 22.750 |
| Albumen, } | 1.004 |
| Water extract, | 8.006 |
| Alcohol extract, | 1.810 |
| Fat, | 2.887 |
| Chloride sodium, | 5.825 |
| Sulphate soda, | 0.400 |
| Carbonate soda, | 0.198 |
| Phosphate soda, | 0.080 |
| Phosphate potash, with traces of iron | 0.974 |
| Carbonate of potash, | 0.291 |
| Silica and sulphate of potash, | 0.555 |

Thus, throughout the organized portions of the animal system, all the several constituents are found in each—only varying in quantity in the various portions analysed. The saliva, then, most evidently supplies an important part in the animal economy, by largely entering into the chemistry of digestion, and the formation of the blood. Were this not the case, it would be difficult to account for the large quantity of saliva secreted during the twenty-four hours—swallowed and absorbed—to be re-secreted and purified for its important action as an animal flux, to unite the food and prepare it for the action of the gastric juices, to dissolve it into chyme, chyle, &c. &c.

In its normal condition the saliva is alkaline, and gives off ammonia. The evaporation of a drop of saliva causes a beautiful crystalline arrangement in form to hydrochlorate of ammonia.

I look upon salivary calculus, in all respects, as being analogous to and identical with certain urinary calculi. It partakes of all their physical appearance, consistence, mottled strata, hardness, softness, friableness, &c. &c. Salivary calculus is capricious in selecting spots for its deposit; but most generally is found on the inside of the lower incisors, and mostly on those teeth of imperfect combination of their elementary and physical constituents—for example—in constitutions of strumous or tubercular diathesis. It is rarely found in perfectly organized teeth—except at periods of sickness—of those possessing sound, vigorous constitutions, and healthful secretions.

On these latter teeth, salivary calculus is unique, it is seen forming a semi-circular or crescent-like lines, encircling the necks of the teeth, close to the enamel, under the edge of the gums, where it blends with the laminæ of the bone of the neck and fangs of the teeth. It is very hard and brittle, requiring considerable force to remove it from its adhesion; it fractures with a sharp clicking sound. Its hardness has enabled me to make a tolerable good polish upon its surface. I have found this peculiar "tartar" to be more destructive to the *alveoli* than all the other deposits and vicious secretions of the stomach and mouth, and in proportion is the cause of the greatest loss of sound teeth to the animal economy. It adheres to the neck of the tooth with the greatest tenacity, as it were, and it gradually progresses, insinuating itself down the fang or fangs of the teeth; irritating the buccal absorbents—always most bountifully supplied in this region—to remove the exciting cause of irritation. In place of which—the absorbents act upon the vital parts—*the exposed alveoli*—the gums still irritated, and still striving to protect the alveoli, curls over their edges, leaving bare the necks of the teeth, and so the process continues, until the bony sockets, or support of the teeth, are entirely removed, and the teeth are left completely denuded and exposed.

This is popularly termed "scurvy in the gums." Many of my professional brethren too, treat it as such. This deposit of the saliva I have seen insinuate itself gradually down to the apex and into the dental nerve foramen of the tooth, and there so irritate the nerve, as to cause the most intense neuralgia along the branches of the fifth pair of nerves. I have seen the dental nervous twig that entered the foramen, leading into the nervous canal of the tooth, severed by this tartar, and I have also seen the tartar insinuate itself into the foramen itself. The dento-alveolar periosteum being pressed upon and pushed from the neck of the tooth by this concrete deposit, as I have said, carrying by absorption the alveoli with it; the teeth, in a perfectly sound state, fall from their positions. Gastric acids act upon the dento-alveolar periosteum precisely in the same manner; that it is not unusual to see teeth drop from the mouth in a perfect, clean and sound state. Hence, in many cases, where the gastric acids have alternately acted upon the surface of the teeth-fangs, with the salivary calculus deposits, the concretion has penetrated so deeply, perfectly, and densely, into the newly made pores, as it were, of the bone, as to require the removal of a portion of the surface of the bone, to relieve it from its embrace. Microscopic examination discovers this calculus to exist in a crystalline state.

The next form of salivary calculus is the soft, friable, sand-like concretion—while the oxalate of lime deposit is partially characterized as above, we here discover two kinds —the simple phosphate of lime, and the ammoniaco-magnesia-phosphate of lime, with the usual compound mixture of animal and extraneous matter.

Salivary calculus is never deposited on the flesh; but only upon such substances as represent the teeth or the nuclei, as in the bladder that offer a surface to receive the deposition of the calculus upon them. Hence, salivary concretions are found deposited on artificial bone or porcelain teeth; on the gold and silver or "continuous gum" plates to which the teeth are attached; to the living and dead fangs of teeth; *but never in or upon the alveolar processes.* The activity of the excited absorbents connected with the gums, embrace the alveoli, and gradually absorb them away.

Salivary calculus is sometimes found in the *Whartonian*

*duct*, in one form known as *ranula.* It has been extirpated from this *duct* as large as a hazel nut. A singular present made to the Anatomical Society, in Paris, by M. Robert, exhibits a hog's bristle, (forced into the duct by a shoemaker,) densely covered with a hard salivary concretion.

As the analysis of the teeth, above, shows that the teeth are alkaline in their chemical combinations, or, in other words, are *neutral*, so the saliva is composed of the same constituents, and is also alkaline. This is a wise provision; upon it the safety and health of the teeth alone depend. We find, however, destructive agents present, and almost always ready to make an onslaught upon the teeth. These agents present themselves in the form of *gastric acids;* the two most powerful of which, and between which and the limy constituents of the teeth, the greatest possible affinity exists, are the phosphoric and oxalic acids. When present, these acids act immediately, slowly and persistingly in combining with and softening the teeth, causing what is termed decay—dry rot; the softening of the teeth, and as is sometimes seen, the actual burning, as it were, of the tooth, as if intense heat had been applied to the edges of the enamel round the decay. I had reduced it to its original elementary *lime.* Thus, between health and sickness, the normal and abnormal condition of the gastric secretions and generations, the teeth are found, by the dentist, remaining either in a sound condition, irritable, painful or otherwise, or slowly or quickly progressing, with chemical decomposition of a portion or all the substance of the tooth or teeth. In this manner the teeth remain healthy, or they are gradually being destroyed Thus, then, is salivary calculus an important element in the natural economy of the system—and it is thus formed, if phosphoric or oxalic or any other acid be generated in the stomach in excess; it is forced by certain actions—laws—into the mouth. Here they meet the lime held in solution and immensely poured forth in the saliva; they chemically unite with these lime solutions, and form, with them, neutral substances in the form of phosphate or oxalate of lime, phosphate of soda, &c., &c., in accordance with the acid presenting itself. A great portion of this "tartar" is swallowed, and the rest adheres to the teeth, which to a great extent it protects, by its presence from the further onslaughts of the acids, which, no doubt, was its original intention. As we often find the very cough, which is the effort of nature to expel and relieve, an irritating viscid phlegm from the mucous lining of the bronchia, is in its turn the chief cause of irritation and mischief, so we find, with regard to the teeth, the good intentions of nature subverted; the efforts of nature to protect the teeth from the acids, by the salivary neutral deposits, by those very deposits, cause the ruin of that she would preserve.

Salivary calculus, then, will often be found, so far from injuring the teeth, to preserve them, unless the acids are in *excess*—when the peculiar action upon the teeth, alveoli and periosteum, are excited or superinduced, causing the loosening and their falling from the jaws.

The soft, friable muco and muco-puriform salivary deposits, will more often be found on these teeth of imperfect formation or combination of their constituents, such as chalk, white or chalky-yellow teeth; on transparent white and transparent yellow teeth, and particularly in these *mottled* teeth where the deposits, during their formation, had been interrupted.

In a strumous or phthisical diathesis, where the teeth are found of pearly beauty and almost painful regularity, or the contrary—as the two extremes are to be as often observed in such diathesis, where the teeth are large, malformed, irregular, crowded or overlapping each other—the peculiar cachexia increases the exhalations of these acids, which in their turn stimulate and excite the salivary glands to pour out its alkaline matter in larger quantity—hence an enormous quantity of salivary concretion is deposited. Again—as in *mercurial, quinine* or acid diathesis, (and sometimes acid saliva accompanies gouty or rheumatic diathesis,) then we find an acidified, purulent, viscid salivary deposit eating away the substance of the teeth, with the greatest rapidity—rendering them, at the same time, exquisitely painful to the touch, whether of the finger nail, a quill or metal tooth-pick.

Again—the saliva deposits an acidified, greasy, viscid greenish matter on the teeth of strumous and rickety children, which soon blackens the substance of the teeth, and corrodes them to the gums. This is also to be observed in children whose parent's constitution partake of a consumptive diathesis. I know of no deposit upon the teeth so acrid, destructive and painful, as this acidified green matter. It is often the cause of local and great constitutional irritation, irritating the origin of the nerves in the sensorium and the recurrent or reflex action upon the nerves of respiration—the par vagum and its recurrent branch, dyspnœa, short or quick breathing, and all the concomitants attending affections of the nervous system, are exhibited. How often *hydrocephalus* is superinduced in children, from this cause, would be impossible even for mental speculation to elucidate.

In mothers of a strumous or phthisical diathesis, whilst nursing; and even during gestation, acid tartar is deposited in large quantities, eating its way under the gums into the necks of the teeth, and destroying them. It is a common observation made by mothers, that they "lost" one or more teeth at each period of gestation. In addition to this increased deposit in mothers nursing, they find the debility or want of "*tone*," *vis visæ*, exhibited by numerous canker sores on the gums and on the inside of the cheeks and lips.

Would your limits permit, I believe that I could carry out this subject to an interesting extent. I will, therefore, conclude this paper on the subject of cleanliness. While I admit the importance of cleanliness to the animal economy, I am free to admit that it does not preserve the teeth from chemical decomposition—*i. e.*, "decay." Cleanliness is important thus far. It gives tone and vigor to the gums, causing them closely to embrace the necks of the teeth, which protects the necks from the lodgments of acids and tartar deposit, and it whitens the teeth. Still the dentist finds daily visits made to him by persons and youth, who cleanse their teeth after every meal, morning and at night, asking professional aid; and so the wear and tear and natural chemical decomposition of the teeth, proceed until artificial substitutes supply their place. Why is this so? Because the system of our population is in a cachectic state. There is no stamina arising out of causes which the subject in question makes inadmissible here. Compare the well educated, the refined, the well living population of our own country with the thousands, nay millions, of the masses of ignorance, filth and degradation visiting our shores—observe the teeth of these emigrants who are as ignorant of, and unacquainted with, dyspepsia and indigestion, as they are the use and appliance of the teeth brush. Observe these people with a thirty to sixty years of encrustation, hiding, nay actually burying under over a half century's accumulated filth, the finest and most perfect teeth that ever ornamented "the human face divine." These people know not neuralgic affliction. All they know in regard to their teeth, is their instructive application.

NEW YORK, *March* 17, 1858.

